# Development of emodepside as a possible adulticidal treatment for human onchocerciasis—The fruit of a successful industrial–academic collaboration

**DOI:** 10.1371/journal.ppat.1009682

**Published:** 2021-07-22

**Authors:** Jürgen Krücken, Lindy Holden-Dye, Jennifer Keiser, Roger K. Prichard, Simon Townson, Benjamin L. Makepeace, Marc P. Hübner, Steffen R. Hahnel, Ivan Scandale, Achim Harder, Daniel Kulke

**Affiliations:** 1 Institute for Parasitology and Tropical Veterinary Medicine, Freie Universität Berlin, Berlin, Germany; 2 School of Biological Sciences, Institute for Life Sciences, University of Southampton, Southampton, United Kingdom; 3 Department of Medical Parasitology and Infection Biology, Swiss Tropical and Public Health Institute, Basel, Switzerland; 4 University of Basel, Basel, Switzerland; 5 Institute of Parasitology, McGill University, Sainte Anne-de-Bellevue, Quebec, Canada; 6 The Griffin Institute, Northwick Park and St. Mark’s Hospital, Harrow, United Kingdom; 7 Institute of Infection, Veterinary & Ecological Sciences, University of Liverpool, Liverpool, United Kingdom; 8 Institute for Medical Microbiology, Immunology and Parasitology, University Hospital Bonn, Bonn, Germany; 9 German Center for Infection Research (DZIF), partner site Bonn-Cologne, Bonn, Germany; 10 Elanco Animal Health, Research & Exploratory Development, Monheim, Germany; 11 Drugs for Neglected Disease initiative, Geneva, Switzerland; 12 Independent Researcher, Cologne, Germany; 13 Department of Biomedical Sciences, Iowa State University, Ames, Iowa, United States of America; University of Pennsylvania, UNITED STATES

## Abstract

Current mass drug administration (MDA) programs for the treatment of human river blindness (onchocerciasis) caused by the filarial worm *Onchocerca volvulus* rely on ivermectin, an anthelmintic originally developed for animal health. These treatments are primarily directed against migrating microfilariae and also suppress fecundity for several months, but fail to eliminate adult *O*. *volvulus*. Therefore, elimination programs need time frames of decades, well exceeding the life span of adult worms. The situation is worsened by decreased ivermectin efficacy after long-term therapy. To improve treatment options against onchocerciasis, a drug development candidate should ideally kill or irreversibly sterilize adult worms. Emodepside is a broad-spectrum anthelmintic used for the treatment of parasitic nematodes in cats and dogs (Profender and Procox). Our current knowledge of the pharmacology of emodepside is the result of more than 2 decades of intensive collaborative research between academia and the pharmaceutical industry. Emodepside has a novel mode of action with a broad spectrum of activity, including against extraintestinal nematode stages such as migrating larvae or macrofilariae. Therefore, emodepside is considered to be among the most promising candidates for evaluation as an adulticide treatment against onchocerciasis. Consequently, in 2014, Bayer and the Drugs for Neglected Diseases initiative (DNDi) started a collaboration to develop emodepside for the treatment of patients suffering from the disease. Macrofilaricidal activity has been demonstrated in various models, including *Onchocerca ochengi* in cattle, the parasite most closely related to *O*. *volvulus*. Emodepside has now successfully passed Phase I clinical trials, and a Phase II study is planned. This Bayer–DNDi partnership is an outstanding example of “One World Health,” in which experience gained in veterinary science and drug development is translated to human health and leads to improved tools to combat neglected tropical diseases (NTDs) and shorten development pathways and timelines in an otherwise neglected area.

## Author summary

*Onchocerca volvulus* is the causative agent of human river blindness, and current elimination programs rely on the use of ivermectin to kill microfilariae. Since no adulticidal drug is available and adult worms have a life span of up to 15 years, elimination programs need to be sustained over several decades. Emodepside is an anthelmintic that is licensed as a dewormer for cats and dogs. Due to its ability to eliminate nematodes located in various extraintestinal host tissues, including migrating larvae and adult filarial worms, it is considered to be an excellent candidate for the treatment of onchocerciasis. Intense collaboration between academia and the pharmaceutical industry has led to a deep understanding of the novel mode of action of the drug and of its parasite target spectrum. Phase I clinical trials with emodepside have demonstrated its safety and adulticide activity against the closely related cattle parasite *Onchocerca ochengi*. Currently, Phase II clinical trials are planned to confirm that emodepside, developed initially to improve animal health, has also the potential to improve human health by tackling a very important neglected tropical disease (NTD).

## Elimination of onchocerciasis—Current status and the need for novel treatment strategies

Onchocerciasis, also known as river blindness, is a parasitic, vector-borne disease caused by the filarial nematode *Onchocerca volvulus*. Filarial diseases such as onchocerciasis and lymphatic filariasis place a tremendous burden on society, with more than 1 billion of the world’s poorest populations at risk of infection [1,2]. Despite *O*. *volvulus* being one of the most prevalent species (see S1 Fig for global distribution), with 21 million people infected and approximately 90 million people at risk of infection in sub-Saharan Africa, plus 14.6 million people suffering from skin disease and approximately 0.77 million people debilitated by severe vision impairment or blindness, its elimination is considered to be possible [3–6].

Four life stages of *O*. *volvulus* live in humans. Infective third-stage larvae (L3) transmitted by the vector (i.e., a species of the genus *Simulium* [black flies]) undergo 2 molts to develop via fourth-stage larvae (L4) into juvenile adults. These mature to become reproductively competent adults (male and female macrofilariae) within around 1 year and have an estimated mean reproductive life span of up to 15 years [7]. Macrofilariae reside primarily in subcutaneous and deep tissue nodules and produce progeny (microfilariae) that live for approximately 1 year, predominantly in the subepidermal layer of the dermis. During the blood meal, female *Simulium* take up microfilariae, which then develop via second-stage larvae (L2) into infective L3 in the insect’s thoracic muscles to complete the life cycle of the parasite [8] (see S2 Fig for illustration of the life cycle).

Onchocerciasis causes severe itching, disfiguring skin lesions, and depigmentation, along with musculoskeletal pain, reduced body mass index, and decreased work productivity [9,10]. The most severe complications attributed to onchocerciasis are severe visual impairment in up to 500,000 cases, blindness in about 270,000 cases, and reduced life expectancy [6,11], although these figures are considered to underestimate the true magnitude of the socioeconomic burden of onchocerciasis [9]. The disease is the second most frequent cause of infectious blindness after trachoma, with almost all cases seen in sub-Saharan Africa [3].

Mass drug administration (MDA) is a means of delivering safe and inexpensive essential medicines based on the principles of preventive chemotherapy, in which populations or subpopulations are offered treatment without individual diagnoses. MDA in endemic areas aims to prevent and alleviate symptoms and morbidity on the one hand and reduce transmission on the other, together improving global health. At present, MDA with ivermectin (Mectizan, Merck, Kenilworth, New Jersey, USA) is the current measure to achieve elimination of onchocerciasis [12–14]. Since ivermectin does not kill the adult worms, it has to be given once or twice per year and is contraindicated in individuals harboring high numbers of *Loa loa* microfilariae due to the risk of life-threatening adverse events [15–17].

Insufficient coverage is one of the main factors hindering progress toward elimination. Reasons for this are many, including difficult to treat areas (*L*. *loa* coinfection, ongoing conflicts), lack of financial resources, and inadequate political engagement. Additionally, program fatigue in MDA has been reported after years of implementation, and an individual in an endemic area may find repeated MDA inconvenient or lose confidence in the MDA campaign. Furthermore, alarming reports of reduced drug efficacy in Ghana and Cameroon may also signal the development of resistance [18,19]. This situation, coupled with the risk of ivermectin resistance, presents an urgent need for a novel anthelmintic drug that preferably possesses macrofilaricidal or permanent sterilizing activity.

Drug development is handicapped by high attrition rates, and many promising molecules fail during preclinical development or in subsequent toxicological, safety, and efficacy testing; thus, research and development (R&D) costs in aggregate are very high. The level of investment into R&D for new products for neglected tropical diseases (NTDs), as reported in the annual Global Funding of Innovation for Neglected Diseases (G-FINDER) surveys, suggests that few NTD areas receive anywhere near the level of funding required; moreover, that funding, when it is available, is rarely allocated in a manner likely to move products through the pipeline to patients [20]. Therefore, no dedicated drug development pipeline for human filariasis is in place, and it is thus essential that stakeholders, funders, industry, academics, and nongovernmental organizations adopt a cooperative approach, sharing responsibility to reduce risks and overcome existing obstacles. Joint efforts should also be made to cut the cost of R&D for new drugs for NTDs.

**Fig 1:**
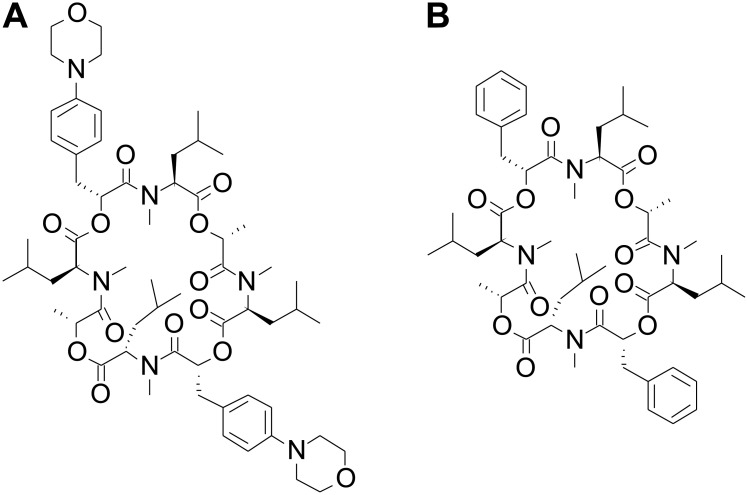
Chemical structures of the semisynthetic emodepside (A) and its precursor PF1022A (B) purified from *Rosellinia* spp. PF1022.

The semisynthetic anthelminthic emodepside (synonyms: PF1022-221, BAY 44–4400, or bismorpholino–cyclooctadepsipeptide) and its parent fermentation product PF1022A are members of the N-methylated cyclooctadepsipeptides (Fig 1). Emodepside is characterized by 2 morpholine rings in para-position of each of the 2 (R)-phenyllactic acids (Fig 1A), which increase solubility and improve bioavailability in comparison to its natural precursor PF1022A (Fig 1B) [21]. This precursor is obtained from a fungus (*Rosellinia* sp. PF1022), which is part of the microflora of the leaves of *Camellia japonica* [22]. Emodepside is registered as a combination product with praziquantel (Profender) for the treatment of cats and dogs infected with, or at risk of, infection with nematodes and cestodes and as a combination product with toltrazuril (Procox) for the treatment of puppies with demonstrated or suspected infection with nematodes and coccidia.

Emodepside has been shown to be microfilaricidal, as well as macrofilaricidal, and has a unique mechanism of action relative to all other anthelmintic drugs [22,23]. The advantage of emodepside over ivermectin is that it targets multiple life cycle stages of filariae, including the adult worms [24]. In the case of the genus *Onchocerca*, the in vitro motility of macrofilariae is affected at lower concentrations than that of the microfilariae [24]. Targeting macrofilariae is expected to result in a reduction in the number of MDA treatment cycles required to break transmission and cure patients of both migrating microfilariae as well as macrofilariae.

Since standard treatment with ivermectin is contraindicated in patients coinfected with *L*. *loa*, availability of an anthelmintic that can be used in regions where loiasis and onchocerciasis are co-endemic would add considerable value toward the goal of eliminating onchocerciasis [15–17]. As to date, emodepside has not been given to patients infected with *O*. *volvulus*, there is obviously no data on patients being coinfected with *L*. *loa*. Thus, after determining the emodepside efficacy and safety profile in patients infected with *O*. *volvulus*, a next step might be to test it in patients coinfected with *L*. *loa* (low microfilariae level) to evaluate the potential benefit in those patients.

The in vivo anthelmintic efficacy of the fermentation product PF1022A was originally demonstrated against the gastrointestinal nematode *Ascaridia galli* in chickens in 1992 [25], followed by reports of anthelminthic effects against a wide range of gastrointestinal nematodes in rats [26–28], mice [29–31], jirds [32], dogs, horses, sheep, and cattle [27]. In addition, emodepside showed efficacy against further species of parasitic nematodes in a variety of hosts, including cerebral and other extraintestinal life cycle stages [22,33–41]. Emodepside has an excellent safety profile, with neurotoxicological side effects only reported rarely in dogs with the multidrug resistance 1 (*mdr-1*) mutation [42]. More importantly, it has been shown to have anthelmintic resistance–breaking properties against several nematode isolates resistant to closantel, fenbantel, fenbendazole, levamisole, and ivermectin in sheep and cattle and against a multidrug-resistant *Ancylostoma caninum* isolate in dogs [43,44].

In addition to its well-known use as an anthelmintic in cats and dogs, the microfilaricidal and macrofilaricidal effects of emodepside have led to the compound being considered as a promising drug development candidate for the treatment of human helminth diseases, including onchocerciasis [22,45,46]. As a result, emodepside is currently being evaluated for the treatment of human onchocerciasis within the scope of a drug development partnership between the Drugs for Neglected Diseases initiative (DNDi) and Bayer [47]. Phase I clinical trials have already been completed successfully [48], and a Phase II trial is planned in 2021.

Here, we outline the development of emodepside for human onchocerciasis, review the elucidation of its novel mode of action and its efficacy against filarial nematodes, both in vitro and in vivo, and describe the initial studies with this encouraging agent in humans.

## Emodepside—Novel mode of action and broad-spectrum activity

### History of the cyclooctadepsipeptide anthelmintics

**Fig 2:**
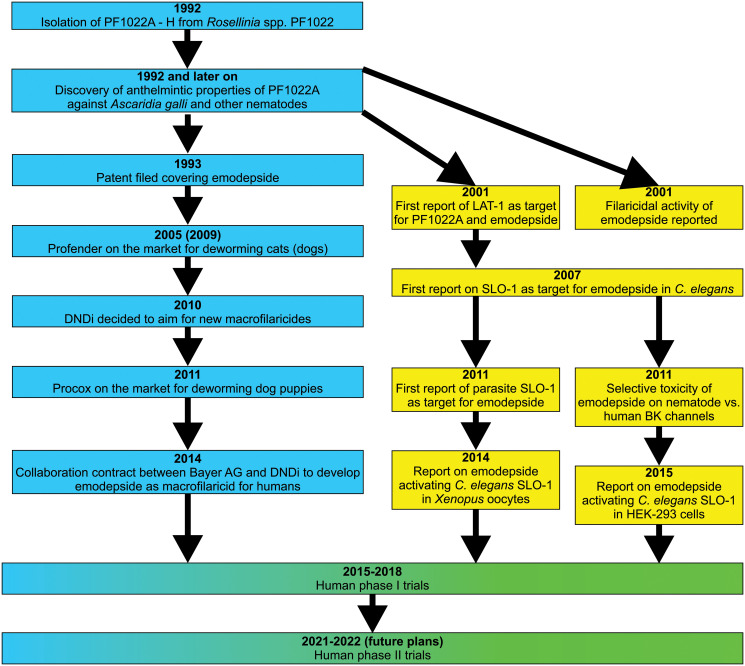
Flow chart summarizing the most important achievements from the isolation of PF1022A to recent and future human trials. Milestones with pharmaceutical industry in lead are shown in blue, and milestones achieved with academia in lead are shown in yellow. BK, Big Potassium; DNDi, Drugs for Neglected Diseases initiative; LAT-1, latrophilin-like receptor.

A flow diagram presenting the most important steps leading from the isolation of PF1022A to human trials evaluating safety and efficacy of the drug is available in Fig 2. In 1992, researchers from Meiji Seika Kaisha (Tokyo, Japan) isolated a new family of N-methylated cyclooctadepsipeptides [49]. The fungus *Rosellinia* spp. PF1022 produces 8 different cyclooctadepsipeptides, designated PF1022A, B, C, D, E, F, G, and H, although PF1022A is produced in the highest amounts by far [50]. PF1022A is also the most potent anthelmintic among the PF1022 members; its anthelmintic activities have been summarized in a previous review [22].

Since the efficacy of the parent compound PF1022A is largely limited to intestinal nematode stages in different host species, further research was focused on identifying cyclooctadepsipeptides with efficacy against extraintestinal stages (including blood and tissue stages). For this purpose, a screening program involving Bayer Animal Health (Leverkusen, Germany), Bayer Crop Science (Monheim, Germany), Meiji Seika Kaisha, and Fujisawa Pharmaceutical (Japan) was initiated. A patent application was subsequently filed by Fujisawa Pharmaceutical for a bis-morpholino derivative of PF1022A, which was later named emodepside [22].

## Elucidation of the mode of action

### Ionotropic γ-aminobutyric acid receptors

Initial studies investigating the mechanism of action of PF1022A found high binding affinity of the cyclooctadepsipeptide to *Ascaris suum* cell membranes, suggesting that there are specific PF1022A binding sites in the membrane preparations that are responsible for its nematocidal activity [51]. PF1022A was also shown to displace radioactive γ-aminobutyric acid (GABA) from *A*. *suum* muscle cells, suggesting an interaction of PF1022A with ionotropic GABA_A_ receptors [52]. However, PF1022A does not appear to act like a GABA agonist in *A*. *suum* muscle cells since it does not produce an increase in muscle membrane conductance similar to GABA [53,54]. Nevertheless, a loss-of-function mutation of the only GABA_A_ receptor gene *unc-49* in the *Caenorhabditis elegans* genome has been shown to cause decreased susceptibility to emodepside [55].

### Latrophilin receptors

The first target of cyclooctadepsipeptides identified in nematodes was the *Haemonchus contortus* latrophilin-like receptor (LAT-1) [56]. An affinity screen of an *H*. *contortus* cDNA expression library identified a G protein–coupled receptor (GPCR) that bound PF1022A [56]. Expression of the full-length *H*. *contortus* LAT-1 in HEK293 cells further supported its potential to act as a receptor for latrotoxin since it initiated downstream Ca^2+^ influx through Cd^2+^ and nifedipine blockable channels (L-type Ca^2+^ channels). Moreover, emodepside, but not its anthelmintically inactive enantiomer, decreased the response of the *H*. *contortus* LAT-1 receptor to latrotoxin [56].

In *C*. *elegans*, simultaneous deletion of both latrophilin paralogs, *lat-1* and *lat-2*, decreased the susceptibility of the pharynx to emodepside [57]. In contrast to *lat-1*, deletion of components of the signaling cascade downstream of the LAT-1 receptor, such as *egl-30* and *egl-8*, causes a decrease in emodepside susceptibility of both pharynx and body wall muscles, yet an *egl-30* gain-of-function mutant increases susceptibility [58]. The impact of defects downstream of LAT-1 on emodepside activity on the pharynx were always more severe than effects of LAT-1 [58]. This suggests that emodepside can directly exert its effects through LAT-1 on the pharynx, with upstream GPCRs augmenting emodepside’s effects on body wall muscles.

Expression patterns of *lat-1* were determined in *C*. *elegans* using a fluorescent reporter gene construct [44,45,47]. In larvae, the promotor was active in neuronal and muscle cells around the pharynx. In contrast, promotor activity ceased in adult worms (which show the highest susceptibility to emodepside) and was limited to neuronal cells [23,59,60]. In contrast, Buxton and colleagues [61] showed expression of *lat-1* mRNA in muscle flaps of *A*. *suum* containing both muscle cells and axons of neuronal cells present in the dorsal and ventral cords. It currently remains unclear if these contrasting results are due to differences between nematode species or the different sensitivities of the methods used. It is also uncertain if emodepside is able to directly modulate LAT-1 from ascarids, since sequence identity between *A*. *suum* and *C*. *elegans* proteins is only 38% (54% similarity) [61].

**Fig 3:**
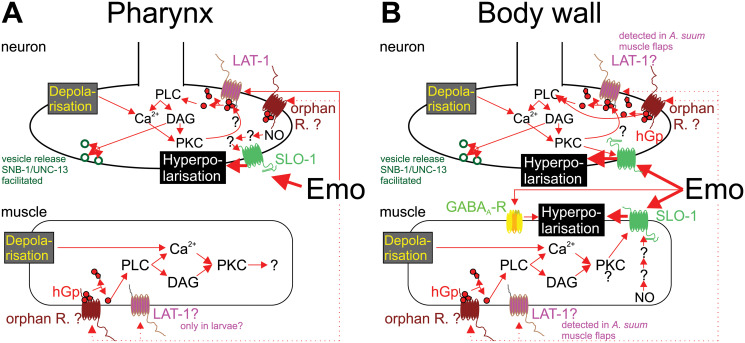
Graphical summary describing the signaling pathways potentially involved in Emo effects in nematodes. Signaling pathways are shown separately for pharynx **(A)** and body wall muscles **(B)**. In several cases, there is no clear or unequivocal evidence for the presence/activity of the signaling component in the particular compartment. In these cases, question marks and dotted lines indicate these uncertainties. Emo directly interacts with the ionotropic GABA_A_-R UNC-49 [52], with LAT-1 [56] and with the voltage-gated Ca^2+^-activated potassium channel slowpoke big K^+^ conductance channel (SLO-1) [59,60]. However, *Caenorhabditis elegans* lines with loss-of-function mutations in *unc-49*, *lat-1*, or both *lat-1* and *lat-2* genes display only modest decreases in their Emo sensitivity [55,57,58]. In contrast, loss of SLO-1 function in *C*. *elegans* leads to complete loss of Emo efficacy, which can be restored by expression of SLO-1 from parasitic nematodes, but only partially by the human orthologue [23,62,63]. This shows that activation of SLO-1 [59,60] is the major effect leading to Emo-induced paralysis as indicated by bold arrows. Activation of SLO-1 or GABA_A_-R UNC-49 can lead directly to membrane hyperpolarization, and, thus, to reduced excitation. UNC-49 is only expressed on body wall muscles (postsynaptic side) [64]. In contrast, SLO-1 channels can be found on presynaptic nerves innervating both pharynx and body wall muscles, as well as on body wall muscles themselves, being absent from pharyngeal muscles [63]. LAT-1 (and potentially other, unidentified GPCRs [orphan receptors]) is assumed to exert its effects via a signal transduction cascade involving an hGp containing the EGL-30 Gqa subunit, leading to activation of the PLC EGL-8 [56–58]. PLC activity hydrolyses PIP2 to DAG and IP3. The latter leads to release of Ca^2+^ from intracellular stores. DAG and Ca^2+^ are both required to activate PKC-β, which is known to be able to modulate SLO-1 responsiveness [61]. On the presynaptic side, DAG is further known to facilitate release of vesicles via the synaptobrevin SNB-1 and UNC-13, a protein essential for presynaptic vesicle release [57]. In *C*. *elegans*, these signaling pathways are also active in presynaptic neurons and postsynaptic body wall muscle cells [58]. In parasitic nematodes, the situation may be different since *lat-1* mRNA was detected in *Ascaris suum* body wall flaps, suggesting that the receptor is expressed in motor neurons and/or body wall muscles [61]. Moreover, NO has been shown to increase potassium currents induced by Emo in *A*. *suum* muscle preparations [61]. It is currently unclear how NO exerts its effect since nematode soluble guanylate cyclases, in contrast to the mammalian enzymes, are not activated by NO [65]. DAG, diacyl glycerol; Emo, Emodepside; GABA_A_-R, γ-aminobutyric acid A receptor; GPCR, G protein–coupled receptor; hGp, heterotrimeric G protein; IP3, inositol 1,4,5-trisphosphate; LAT-1, latrophilin-1; NO, nitric oxide; PIP2, phosphatidylinositol 4,5-bisphosphate; PKC-β, protein kinase C β; PLC, phospholipase C; SLO-1, slowpoke big K^+^ conductance channel.

In terms of understanding the mode of action of emodepside, it is important to note that *C*. *elegans* with double deletion of *lat-1* and *lat-2* are still nonviable in emodepside [58], suggesting that while latrophilin is a target of emodepside, other targets must also be key to its anthelmintic action. Knowledge regarding the different pathways involved in the effects of emodepside in nematode neurons and muscle cells is graphically summarized in Fig 3.

### Voltage-gated and Ca^2+^-activated potassium channels (SLO-1)

**Fig 4:**
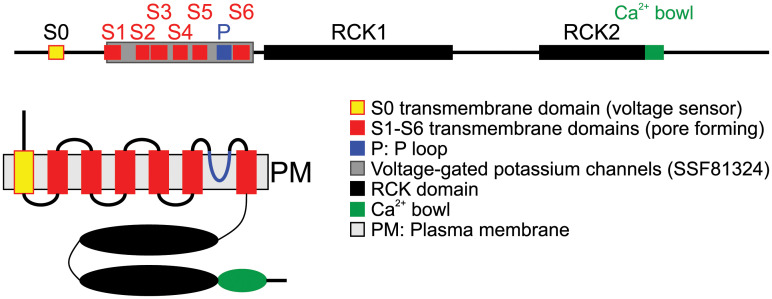
Schematic drawings showing domain composition (A) and topology in the plasma membrane of a SLO-1 subunit (B). The scheme in **(A)** is drawn to scale based on *Onchocerca volvulus* SLO-1a (GenBank accession number: MW039265; the protein contains 7 transmembrane domains. The transmembrane domains S2–S4 contain charged amino acids and function as voltage sensor, while S5 and S6 are involved in building the pore. For this purpose, 4 subunits form a tetramer. The P loop is involved in tetramer formation and pore building. The 2 RCK domains and the Ca^2+^ bowl are involved in regulation of conductance by intracellular Ca^2+^ levels. P loop, pore-forming loop; PM, plasma membrane; RCK, regulator of potassium conductance; SLO-1, slowpoke big K^+^ conductance channel.

In order to determine the molecular mediators of the latrophilin-independent effect of emodepside, a genetic screen in *C*. *elegans* revealed resistant worms with mutations in alleles of *slo-1* encoding a calcium-activated potassium channel [23,66]. SLO-1 channels are important for rapid repolarization of cells after depolarization during action potentials; a functional channel is made up of 4 subunits containing 7 transmembrane helices each (Fig 4). A functional null mutant of *slo-1* was found to develop and feed normally in the presence of a concentration of emodepside that impairs development, inhibits feeding, and paralyzes wild-type worms [67]. Interestingly *C*. *elegans* strains carrying *slo-1* gain-of-function mutations are sluggish, immotile, and retain eggs, phenocopying the effect of emodepside on *C*. *elegans* behavior [68]. These observations suggest that emodepside is a SLO-1 agonist, activating potassium currents to inhibit neuromuscular function. This has been confirmed through heterologous expression of *slo-1* in *C*. *elegans* pharyngeal muscle [63], HEK293 cells [60], and *Xenopus* oocytes [59,60].

**Fig 5:**
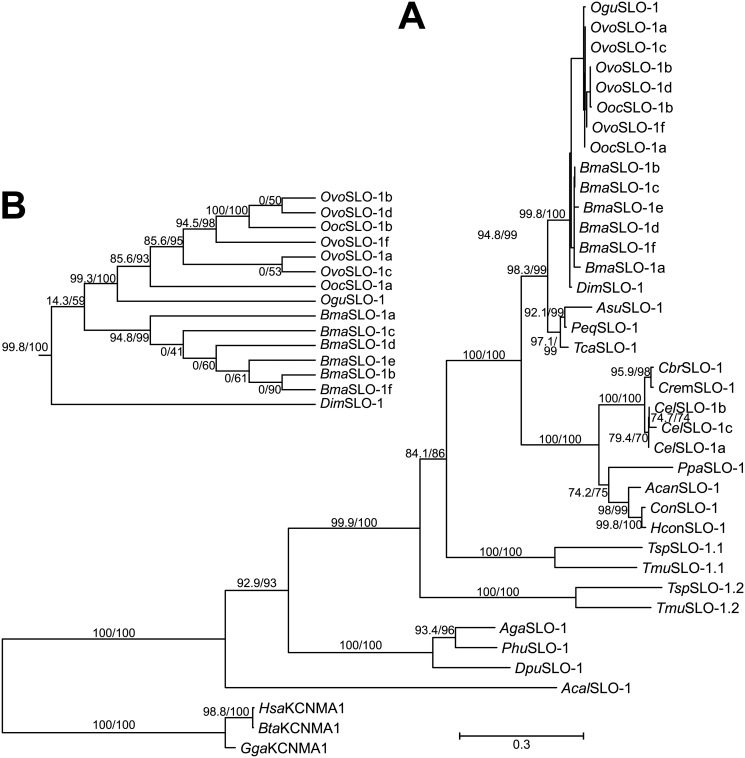
Phylogenetic analysis of nematode SLO-1 channels. **(A)** Maximum likelihood phylogram calculated from full-length Slo-1 proteins. Slo-1 sequences from the nematode species *Cel*, *Cbr*, *Cre*, *Pca*, *Hco*, *Con*, *Acan*, *Ovo*, *Ooc*, *Ogu*, *Bma*, *Dim*, *Tca*, *Peq*, *Asu*, *Tmu*, and *Tsu* were aligned with orthologs from *Dme*, *Aga*, *Phu*, *Dpu*, *Acal*, *Gal*, *Bta*, and *Hsa*, representing an outgroup, using MCoffee [69]. For *Onchocerca* spp. and *Brugia malayi*, all splice variants were included, but only the variants *slo-1a-c* for *Caenorhabditis elegans*. For all other species, only a single splice variant was used per gene. Accession numbers are available in S1 Table. ModelFinder [70] was applied to identify the optimal amino acid substitution model. The VT+G4 amino acid substitution model was used with a Γ shape alpha set to 0.4242 and the 4 substitution relative rate categories (with equal frequencies) of 0.0203, 0.1996, 0.7562, and 3.0239. Maximum likelihood trees were calculated with IQ-TREE [71] using 2,000 iterations of the Shimodaira–Hasegawa modification of the approximate likelihood ratio test (before the slash) and 2,000 ultrafast bootstrapping replicates (behind the slash). The scale bar represents 0.3 substitutions per site. **(B)** Enlarged cladogram showing the relationship of filarial SLO-1 proteins. For clarity, node support values for this part of the tree are only indicated in B. *Acal*, *Aplysia californica*; *Acan*, *Ancylostoma caninum*; *Aga*, *Anopheles gambiae*; *Asu*, *Ascaris suum*; *Bma*, *Brugia malayi*; *Bta*, *Bos taurus*; *Cbr*, *Caenorhabditis briggsae*; *Cel*, *Caenorhabditis elegans*; *Con*, *Cooperia oncophora*; *Cre*, *Caenorhabditis remanei*; *Dim*, *Dirofilaria immitis*; *Dme*, *Drosophila melanogaster*; *Dpu*, *Daphnia pulex*; *Gal*, *Gallus gallus*; *Hco*, *Haemonchus contortus*; *Hsa*, *Homo sapiens*; *Ogu*, *Onchocerca gutturosa*; *Ooc*, *Onchocerca ochengi*; *Ovo*, *Onchocerca volvulus*; *Pca*, *Pristionchus pacificus*; *Peq*, *Parascaris equorum*; *Phu*, *Pediculus humanus humanis*; SLO-1, slowpoke big K^+^ conductance channel; *Tca*, *Toxocara canis*; *Tmu*, *Trichuris muris*; *Tsu*, *Trichuris suis*.

The role of SLO-1 in the toxicity of emodepside to parasitic nematodes of companion animals and livestock was confirmed by the cloning of *slo-1* from *A*. *caninum* and *Cooperia oncophora* and its expression in the *C*. *elegans* functional null *slo-1* mutant, NM1968 *slo-1(js379)V*. Expression of *A*. *caninum* and *C*. *oncophora slo-1* genes in NM1968 was found to confer emodepside sensitivity to this otherwise unresponsive mutant and ameliorate the locomotor deficit of the *C*. *elegans slo-1* null background [62]. This provides strong evidence that emodepside exerts its anthelmintic action in both nematode species (*A*. *caninum* and *C*. *oncophora*) through the activation of SLO-1. The conservation of *slo-1* in the phylum Nematoda provides further evidence that this is the major determinant of its selective anthelmintic action (see Fig 5) [59].

To address the selective toxicity of emodepside in terms of its impact on the mammalian host, the actions of the closest human orthologue of *C*. *elegans slo-1*, *kcnma1*, was investigated. While emodepside was found to be a potent and prolonged activator of *C*. *elegans* SLO-1 heterologously expressed in HEK293 cells, it only caused transient activation of KCNMA1, followed by inhibition [60]. In the same study, emodepside also activated *Drosophila slo-1*, although its ability to modify the rectifying properties of the emodepside-induced current differed from that seen with *C*. *elegans slo-1* [60]. These functional differences in the interaction of emodepside with calcium- and voltage-activated potassium channels provide a mechanistic explanation for its selective anthelmintic action.

A recent study used comparison of *C*. *elegans* wild strains with the N2 Bristol laboratory strain regarding emodepside responsiveness [72]. The authors found that there was substantial natural variation in susceptibility to emodepside. Some but not all of this variation could be explained by variation in the SLO-1 channel. The remaining variation was not explainable by variation in LAT-1, suggesting that there are additional genetic factors contributing to the level of emodepside susceptibility. These might include any of the signal transduction pathway components downstream of LAT-1 identified previously [58] and depicted in Fig 3 or additional, currently unidentified loci.

Taken together, the studies in *C*. *elegans* and parasitic nematodes have identified the calcium- and voltage-activated potassium channel SLO-1 as the major receptor mediating emodepside’s anthelmintic action. The identity and properties of the pharmacophore for emodepside harbored by the channel remain a topic of speculation, although its physicochemical properties and profile of effects suggest a binding site within the membrane at a lipid:protein interface [58]. However, elucidating the interaction of emodepside with SLO-1 was an important step toward understanding the molecular basis of its mode of action.

## Emodepside as a filaricidal agent

There is an urgent need for an orally delivered macrofilaricidal drug suitable for MDA programs. In light of the success of emodepside as a veterinary anthelmintic [22], studies to evaluate the efficacy of this drug against filarial nematodes were planned. Initial studies employed in vivo filarial models in the southern multimammate mouse, *Mastomys coucha* [73]. Here, it was shown that a single dose of emodepside (100 mg/kg) applied as a spot-on formulation was able to substantially suppress microfilaremia in *Acanthocheilonema viteae* and *Litomosoides sigmodontis* infections for at least 100 days and in *Brugia malayi* infections for at least 150 days, when the drug was administered during the maturation of adult worms [73]. Only larval and preadult stages of *A*. *viteae*, and not those of *L*. *sigmodontis* and *B*. *malayi*, were affected by this treatment. A single dose of oral, subcutaneous, or spot-on treatment of 100 mg/kg emodepside has also been shown to eliminate all adult *A*. *viteae* in the same model [74]. In contrast, elimination of adult *L*. *sigmodontis* required repeated oral treatment of 100 mg/kg emodepside on 5 consecutive days, whereas adult *B*. *malayi* were not eliminated by this treatment schedule [74]. Nevertheless, severe pathological alterations in the intrauterine stages of all 3 parasite species were noted at a much lower single dose of emodepside, which is likely to explain the long-term effects of the drug on microfilarial density [73,74]. A direct microfilaricidal effect of emodepside was further shown in mice injected with microfilariae of *Onchocerca lienalis* at doses of ≥5 × 1.56 mg/kg [75].

The abovementioned in vivo studies showed that emodepside clears microfilariae of different filarial species, but has a less pronounced effect on the adult filarial burden. However, in vitro studies using a 4-day assay analyzing the motility of *L*. *sigmodontis* adult male and female worms showed complete inhibition of motility in both sexes at emodepside concentrations as low as 1 × 10^−8^ M. *L*. *sigmodontis* microfilariae and L3 larvae were less susceptible to emodepside, with complete motility inhibition demonstrated in a 4- and 3-day in vitro assay at an IC_50_ of 5 × 10^−9^ M and 3.5 × 10^−7^, respectively. Similarly, for microfilariae of *A*. *viteae* and *B*. *malayi*, complete inhibition of motility in a 4- and 3-day in vitro assay was demonstrated at an IC_50_ of 1.5 × 10^−8^ M and 6.4 × 10^−8^ M, respectively [24].

Further studies used several in vitro and in vivo systems that have been developed to study drug effects on *Onchocerca* spp. and *Brugia* spp. (see [5]). Utilizing these protocols, the efficacy of emodepside against 3 species of filarial parasites was evaluated, examining the effects of drug concentration and duration of exposure on adult male *Onchocerca gutturosa*, adult male and female *Brugia pahangi*, and microfilariae of *O*. *lienalis* in vitro [75]. The effects of drug dosage, formulation and route of administration on the survival of *O*. *lienalis* microfilariae in mice were also assessed [75].

*O*. *gutturosa* adult males obtained from the nuchal ligament connective tissues of naturally infected cattle were tested in a 5-day in vitro assay (concentration range 2.95 × 10^−12^ M to 1.25 × 10^−5^ M). Emodepside immobilized them at a concentration of ≥4.8 × 10^−8^ M, producing an EC_50_ for motility of 9 × 10^−10^ M [35]. However, 3-(4,5-dimethylthiazol-2-yl)-2,5-diphenyltetrazolium bromide (MTT)/formazan colorimetry results demonstrated that emodepside concentrations ≤1.25 × 10^−5^ M had no significant effect on macrofilarial viability, indicating that the worms were paralyzed, but not dead, after 5 days exposure to emodepside. A subsequent long-term in vitro assay confirmed that 5-day exposure to 1.25 × 10^−5^ M emodepside killed (measured both by motility and MTT colorimetry) *O*. *gutturosa* adult males after a total of 40 days of culture, indicating that worms did not recover following the initial 5-day exposure to emodepside [75].

Macrofilaricidal properties of emodepside were also evaluated against adult male and female *B*. *pahangi* in vitro [75]. These parasites were significantly less sensitive to emodepside than *O*. *gutturosa*, with *B*. *pahangi* parasites remaining active at a low level at 1.25 × 10^−5^ M in a 5-day assay, resulting in a sex-dependent motility EC_50_ of 6 × 10^−8^ M for male worms and 4.3 × 10^−7^ M for females. Poor motility was also observed throughout a 10-day trial in which worms were transferred to a drug-free medium on day 5, with a similar partial effect seen on MTT values, indicating a significant drug effect, but not death [75].

Sex-dependent effects of emodepside have also recently been reported for *B*. *malayi* [76]. In particular, male and female worms have been shown to express distinctive splice variants of the emodepside target SLO-1, which differ greatly in their responsiveness to emodepside, presumably accounting for the major differences in emodepside susceptibility between the different sexes in this species.

A summary of studies investigating the activity of emodepside against filaria is provided in Table 1.

**Table 1:** Efficacy studies for emodepside against filaria.

Filaria species	Emodepside efficacy	Reference
**In vitro studies**		
*Litomosoides sigmodontis*	Adult worms: complete motility inhibition (IC_50_ 1 × 10^−8^ M)^a^ L3: complete motility inhibition (IC_50_ 9 × 10^−9^ M)^a^	[24]
*Acanthocheilonema viteae*	MF: complete motility inhibition (IC_50_ 1 × 10^−9^ M)^a^	[24]
*Brugia malayi*	MF: complete motility inhibition (IC_50_ 1 × 10^−9^ M)^a^	[24]
*Onchocerca gutturosa*	Adult male worms: complete motility inhibition (EC_50_ 9 × 10^−10^ M)^b^	[75]
*Brugia pahangi*	Adult male worms: reduced motility (EC_50_ 6 × 10^−8^ M)^b^	[75]
	Adult female worms: reduced motility (EC_50_ 4.3 × 10^−7^ M)^b^	
**In vivo studies in mice**		
*A*. *viteae*	MF: elimination for ≥100 days (100 mg/kg emodepside)	[73]
	Adult worms: elimination within 56 days (100 mg/kg emodepside)	[74]
*L*. *sigmodontis*	MF: elimination for ≥150 days (100 mg/kg emodepside)	[73]
	Adult worms: elimination within 56 days (5 × 100 mg/kg emodepside)	[74]
*B*. *malayi*	Intrauterine stages: severe pathological alterations (25 mg/kg emodepside)	[74]

^a^4-day assay.

^b^4-day assay.

L3, third-stage larvae; MF, microfilariae.

## Development of emodepside for onchocerciasis

### From animal health to neglected tropical diseases

Knowledge of the safety, efficacy, and pharmacokinetics (PK) of pharmaceuticals in animals can allow for a rapid transition into human use. Thus, veterinary anthelmintics should be considered as a priority for use against helminth parasites in humans, where there are still unmet needs [46,77].

Ivermectin is an example of applying the experience of anthelmintics developed for animal health to human NTDs. The drug was developed in the mid-1970s for control of livestock nematodes. By the late 1970s, it was observed that ivermectin was microfilaricidal against *Onchocerca* spp. in horses [78] and cattle [79] and prohibited the development of larval stages of the filarial nematode *Dirofilaria immitis* in dogs [80]. These observations led to the consideration of ivermectin for the control of *O*. *volvulus* [79]. The development of ivermectin for onchocerciasis, and, more recently for lymphatic filariasis, has been a highlight of efforts to control NTDs. For both filarial diseases, control programs rely on the microfilaricidal effect and temporal sterilization of female worms, which inhibit the release of new microfilariae for several months. For onchocerciasis and lymphatic filariasis control and elimination programs, ivermectin is donated under the Mectizan Donation Program established by Merck in 1987 [81].

There are a number of other examples of pharmaceuticals that were first developed for use in animal health ultimately having a huge impact on NTDs. Albendazole and mebendazole were first developed for animal health and have subsequently been used to control human soil-transmitted helminths (STHs) [82]. Hundreds of millions of doses per year of these anthelmintics are now donated for control of STH and lymphatic filariasis (albendazole) in humans. Another example, praziquantel, developed by Bayer and E. Merck (Merck Group), was patented initially as a veterinary drug. Subsequently, it was shown to be highly efficacious against human schistosomes, and it became essential in the fight against NTDs [83].

Bayer has a long history of discovering drugs for the treatment of NTDs. It developed a number of antiprotozoal drugs that are of considerable importance, including the suramin (Naganol) against African trypanosomiasis (discovered in 1924), the antimalarial drug chloroquine (in 1934 to 1937), and nifurtimox against Chagas disease (in 1972) [84]. Bayer-developed anthelmintics include antimosan against *Schistosoma mansoni* (in 1931), Miracil D (lucanthone) against some schistosomes (in 1940), and niclosamide (in 1953), which was used against tapeworms before the discovery of praziquantel [85]. Now, emodepside is the latest Bayer drug entering the field of tropical medicine.

The DNDi, a collaborative, patient needs–driven, not-for-profit R&D organization, was founded in 2003. DNDi’s primary focus is the development of drugs for the treatment of the most neglected diseases around the world [86]. In 2010, DNDi approved the inclusion of filarial diseases in its portfolio aiming to develop a safe, efficacious, affordable, and field-adapted drug targeting macrofilariae against onchocerciasis and/or lymphatic filariasis. One of the strategies pursued was to identify veterinary drugs and evaluate their suitability for development as macrofilaricides for human use [47].

Emodepside was originally identified as a potential macrofilaricide through a drug discovery effort aiming to identify macrofilaricidal drugs for filarial diseases sponsored by WHO Special Programme for Research and Training in Tropical Diseases (WHO-TDR) in the early 2000s [3,75,87]. In 2014, DNDi and Bayer signed an agreement for a collaboration on the development of emodepside as a potential treatment for onchocerciasis [47]. The scope of this agreement was to conduct a gap analysis of the veterinarian safety package as part of the completion of an investigational medicinal product dossier (IMPD). In addition, in vitro and in vivo studies were conducted with *L*. *sigmodontis* [24,88], a surrogate filarial nematode for efficacy against *O*. *volvulus* [89–91]. In the agreement, Bayer committed to conduct pharmaceutical development and supply of the drug, while DNDi was responsible for both nonclinical and clinical development of the molecule.

### The *Onchocerca ochengi*/cattle model for preclinical proof of concept

*O*. *ochengi* is a sibling species to *O*. *volvulus* [92,93] infecting cattle, which is widely used for research into the basic biology of *Onchocerca* spp., drug effects, and vaccine candidates [94]. To assess the predictability of emodepside effects in the *O*. *ochengi* cattle model for human onchocerciasis, a recent study compared the emodepside susceptibility of SLO-1 of both *Onchocerca* species. A comparison of amino acid sequences from different isolated isoforms indicated that SLO-1 is highly conserved between both species (Fig 5). Moreover, when functionally expressed in the *Xenopus* oocyte expression system, *O*. *ochengi* and *O*. *volvulus* SLO-1 isoforms were all able to form homomeric channels, showing comparable levels of emodepside sensitivity [95].

In order to obtain reliable data using the *O*. *ochengi*/cattle model, it is crucial to achieve drug concentrations at the target site that are comparable with those in humans. The maximum tolerated dose (MTD) of emodepside in taurine Holstein cattle was found to be 1 mg/kg, but 0.75 mg/kg was used conservatively in indicine (zebu) cattle in Cameroon to compensate for environmental stresses [96]. Analytical methods were also established to determine emodepside concentrations in skin and nodules containing adult *O*. *ochengi* after administration of different doses showing that an intravenous dose of 0.15 mg/kg in cattle was equivalent to 10 mg in humans, achieving the same local target concentration. Using this model, the density of microfilariae in the skin was not affected by a single 0.15 mg/kg dose of emodepside. In contrast, a single 0.75 mg/kg dose and 0.15 mg/kg given daily for 7 days transiently reduced microfilarial density [97]. In addition, 0.75 mg/kg emodepside given daily for 7 days resulted in an initial reduction, then a transient rise followed by complete clearance, of microfilariae in 4 out of 7 animals within 18 months of treatment. The motility of adult macrofilariae recovered from nodules was significantly affected over time at all emodepside doses for females, but only at the highest dose for males [97]. In 5 out of 7 cattle, including those with no microfilariae, the 7 × 0.75 mg/kg treatment scheme caused death or sterility of female worms, demonstrating slow-acting macrofilaricidal and sterilizing effects [98].

### Emodepside in Phase I human trials

Based on the available preclinical data, permission to proceed with Phase I trials was granted by the United Kingdom Medicines & Healthcare products Regulatory Agency (MHRA), and the study was initiated in December 2015 in the UK and completed in October 2018. Results of Phase I studies indicate that predicted efficacious levels could be reached safely in humans [99].

Phase I clinical trials with emodepside were comprised of single- (NCT02661178) and multiple-dose (NCT03383614) safety, tolerability, and PK studies and a relative bioavailability study (NCT03383523). In the single- (0.1 to 40 mg emodepside) and multiple-dose (5 or 10 mg) studies, an oral liquid formulation of emodepside was used, with the multidose escalation study also comparing emodepside 10 mg daily with 10 mg twice daily. In the bioavailability study, 2 immediate-release (IR) tablet emodepside formulations were compared with the liquid formulation (5 or 10 mg) [48]. As a liquid service formulation, emodepside was rapidly absorbed under fasting conditions, with dose-proportional increases in plasma concentrations at doses from 1 mg to 40 mg. The half-life during the first 24 hours after dosing was around 11 hours, followed by a terminal elimination half-life >500 hours. Emodepside was less bioavailable in the fed state. Emodepside was well tolerated overall, with no major safety concerns. The rate of absorption was slower, and the peak serum concentration (C_max_) was slightly lower with the amorphous solid dispersion tablets than with the liquid service formulation (NCT03383614, NCT02661178, and NCT03383523). These data enabled us to select a field-adapted tablet formulation that will open the way for further clinical development of emodepside in individuals with onchocerciasis. In terms of macrofilaricidal activity, a target concentration corresponding to the in vitro minimum inhibitory concentration against *L*. *sigmodontis* of 100 ng/ml was assumed to be required. Simulation studies were used to model dosing regimens that would achieve such a target site concentration. A 15-mg dose with the gastrosoluble tablet is predicted to provide exposure that will achieve the target concentration for clinical efficacy [48,99].

A Phase II clinical trial will take place in Hohoe, Ghana. The study is a randomized, double-blind, parallel group trial to investigate emodepside in subjects with *O*. *volvulus* infection, comprising 2 parts. In part 1, the safety, tolerability, pharmacodynamics, PK, and dose–response relationship for efficacy (proof of concept) was investigated. In part 2, the efficacy of selected doses, safety, tolerability, and PK will be investigated [100].

## Beyond onchocerciasis—Should emodepside be considered for human soil-transmitted helminths?

STH infections, including *Ascaris lumbricoides*, *Trichuris trichiura*, and the 2 hookworm species *Ancylostoma duodenale* and *Necator americanus*, are highly prevalent and responsible for a considerable public health burden [101]. Chemotherapy is the strategy of choice for treatment and control of these infections. However, there is a need to discover and develop alternative drugs since treatment options are limited, drug resistance is a concern, and, importantly, the 2 most widely used drugs, the benzimidazoles albendazole and mebendazole, have limited activity against *T*. *trichiura* when used in a single-dose regimen [102]. Given the limited drug discovery pipeline for STH infections, veterinary anthelmintics are an attractive starting point for crossover development for the treatment of these diseases, particularly given the fact that almost all drugs against these pathogens have been introduced initially into the veterinary market (see above).

Emodepside could be an excellent drug candidate for the treatment of STH infections. In S2 Table, studies conducted to date investigating the activity of emodepside against *Trichuris* spp., ascarids, *Strongyloides ratti*, and hookworm species have been summarized. Briefly, emodepside is highly active against *T*. *vulpis* in dogs at single oral doses of 0.5 to 2 mg/kg [37]. In the *T*. *muris* mouse model, an ED_95_ of 24.5 mg/kg for oral treatment was calculated [103], while a recent study reported an ED_50_ of 1.2 mg/kg and complete clearance of the parasites at 75 mg/kg for a single oral treatment [104]; for comparison, an ED_50_ of 4.7 mg/kg was recently determined for a single dose of oxantel pamoate against *T*. *muris* in mice [105]. A single dose of emodepside was shown to be highly effective against ascarids in cats (*Toxocara cati* and *Toxascaris leonina*) and dogs (*Toxocara canis* and *T*. *leonina*) [34,38]. Remarkably, it is also able to suppress galactogenic transmission of *T*. *cati* [106]. Studies with emodepside against hookworms were conducted with the mouse and rat roundworms *Heligmosomoides bakeri* and *Nippostrongylus brasiliensis* [107], the zoonotic parasites *Ancylostoma tubaeforme* [37] and *A*. *ceylanicum* [104] in cats and *A*. *caninum* in dogs [39,108], as well as the human parasite *N*. *americanus* in a hamster model [104]. Importantly, emodepside also revealed activity against *Strongyloides*, a neglected helminth species [107].

In summary, based on the preclinical and clinical studies in animals conducted over the past 20 years, it can be anticipated that emodepside would be a useful addition to the small drug armamentarium for human STH infections. As described below, Phase II clinical trials are planned with emodepside against *T*. *trichiura* and hookworm infections. In parallel, confirmatory preclinical studies should take place with the single product against *Strongyloides stercoralis* in dogs and against *Ascaris* spp. in pigs, as well as for migrating larvae in the mouse model.

## Current status of development program and outlook

Using a multiple-dose therapeutic scheme, emodepside will be evaluated for its macrofilaricidal and long-term sterilizing activity in onchocerciasis patients. Emodepside’s activity against *T*. *trichiura* and hookworms will also be evaluated in parallel.

Although much work remains to be done, current progress suggests that emodepside could be a valuable future weapon in the arsenal to fight against important NTDs caused by parasitic nematodes.

## Supporting information

S1 Fig Geographical distribution and status of PC in endemic countries in 2017. PC, preventive chemotherapy.
(JPG)

S2 Fig Life cycle of *Onchocerca volvulus*. Blackflies of the genus *Simulium* transmit L3 of *O*. *volvulus* onto human skin from where larvae then actively penetrate into the bite wound (1). In subcutaneous tissues, larvae develop into adult filariae (2). Adults reside in nodules in subcutaneous connective tissues (3) and can live there for up to 15 years. Nodules can contain multiple relatively shorter male (19–42 mm) and longer female (33–50 cm) worms. Female worms are viviparous and can produce microfilariae for about 9 years. Microfilariae are unsheathed and have a length of 220–360 μm and a diameter of 5–9 μm. They can survive up to 2 years in the human host. Although microfilariae can be found occasionally in peripheral blood, urine, and sputum, they are typically found in the skin and in the lymphatics of connective tissues (4). Transmission to the intermediate host occurs when a blackfly ingests microfilariae during blood feeding (5). Microfilariae penetrate the wall of the blackfly’s midgut and migrate through the hemocoel to the thoracic muscles (6) where the development from first-stage (7) into L2 and L3 (8) occurs. The iL3 migrate to the blackfly’s proboscis (9) and can infect another human when the vector takes a blood meal (1). iL3, third-stage infective larvae; L2, second-stage larvae; L3, third-stage larvae.
(JPG)

S1 Table SLO-1 protein database entries included in the phylogenetic analysis. SLO-1, slowpoke big K^+^ conductance channel.
(DOCX)

S2 Table Efficacy studies for emodepside against *Trichuris* spp., ascarids, hookworms, and *Strongyloides ratti*. (DOCX)
