# Supplementary figures and images for: Development of emodepside as a possible adulticidal treatment for human onchocerciasis—The fruit of a successful industrial–academic collaboration
Source: PLoS Pathog. 2021 Jul 22;17(7):e1009682. doi: 10.1371/journal.ppat.1009682 (PMC8297762; doi:10.1371/journal.ppat.1009682)

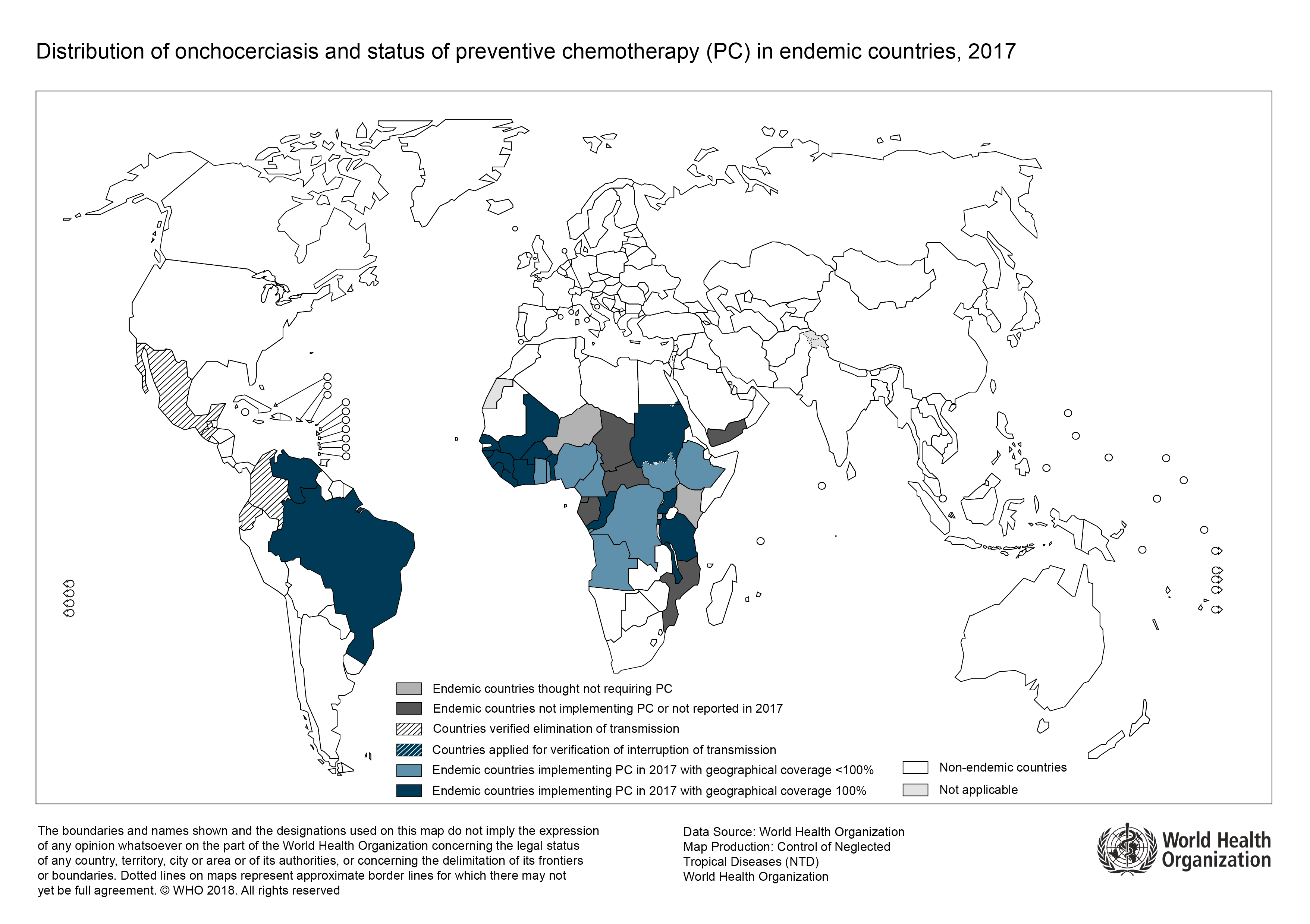

Supplement: S1 Fig — PC, preventive chemotherapy. (JPG) [file ppat.1009682.s001.jpg]

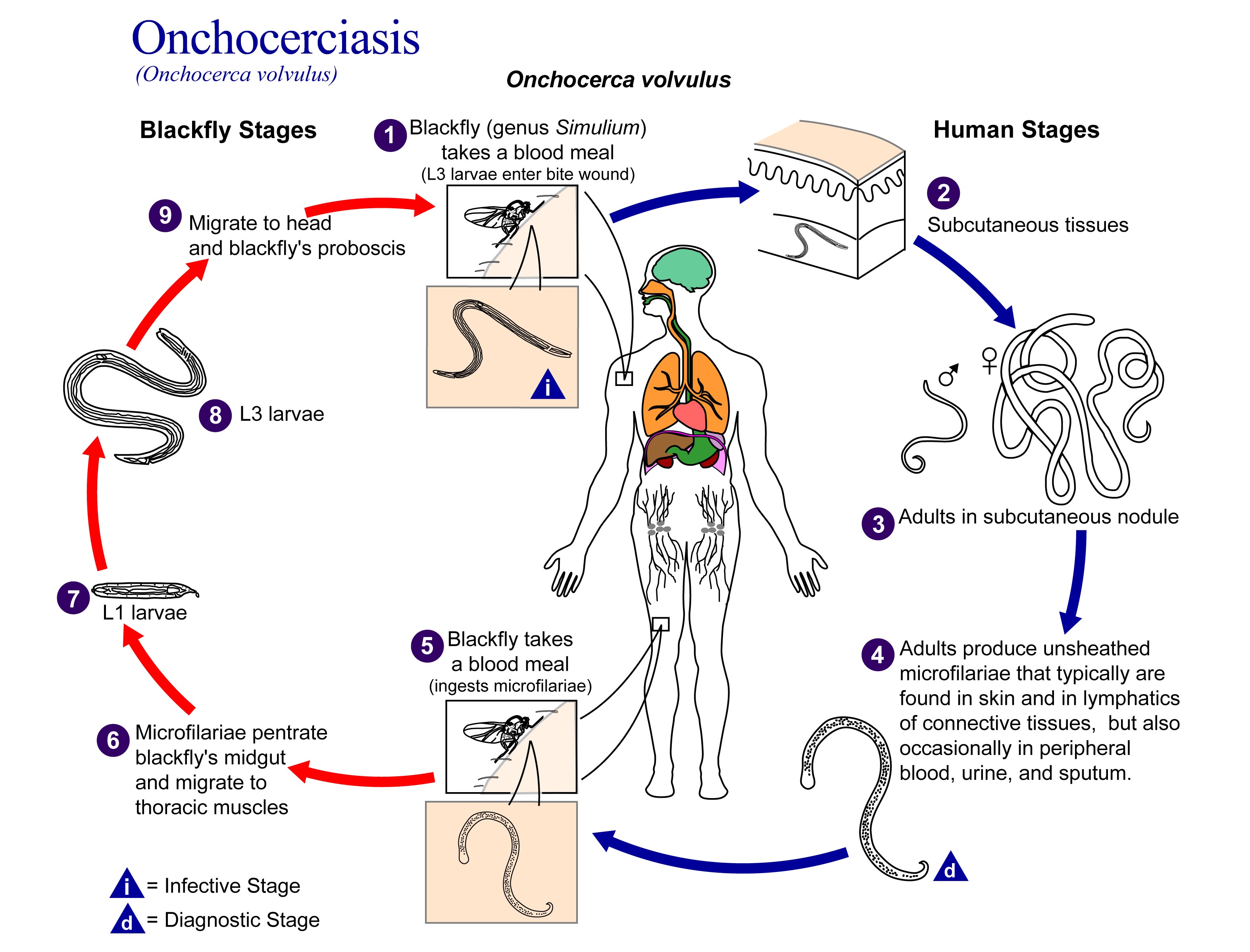

Supplement: S2 Fig — Blackflies of the genus Simulium transmit L3 of O. volvulus onto human skin from where larvae then actively penetrate into the bite wound (1). In subcutaneous tissues, larvae develop into adult filariae (2). Adults reside in nodules in subcutaneous connective tissues (3) and can live there for up to 15 years. Nodules can contain multiple relatively shorter male (19–42 mm) and longer female (33–50 cm) worms. Female worms are viviparous and can produce microfilariae for about 9 years. Microfilariae are unsheathed and have a length of 220–360 μm and a diameter of 5–9 μm. They can survive up to 2 years in the human host. Although microfilariae can be found occasionally in peripheral blood, urine, and sputum, they are typically found in the skin and in the lymphatics of connective tissues (4). Transmission to the intermediate host occurs when a blackfly ingests microfilariae during blood feeding (5). Microfilariae penetrate the wall of the blackfly’s midgut and migrate through the hemocoel to the thoracic muscles (6) where the development from first-stage (7) into L2 and L3 (8) occurs. The iL3 migrate to the blackfly’s proboscis (9) and can infect another human when the vector takes a blood meal (1). iL3, third-stage infective larvae; L2, second-stage larvae; L3, third-stage larvae. (JPG) [file ppat.1009682.s002.jpg]
