# Supplementary material for: Development of emodepside as a possible adulticidal treatment for human onchocerciasis—The fruit of a successful industrial–academic collaboration
Source: PLoS Pathog. 2021 Jul 22;17(7):e1009682. doi: 10.1371/journal.ppat.1009682 (PMC8297762; doi:10.1371/journal.ppat.1009682)
Supplement: S1 Table — SLO-1, slowpoke big K+ conductance channel. (DOCX) [file ppat.1009682.s003.docx]

| Species | Systematic position | Protein | Amino acid length | Molecular  Weight (kDa) | Accession no.^a^ |
| --- | --- | --- | --- | --- | --- |
| *Caenorhabditis elegans* | Chromadorea  Rhabditida  Rhabditoidea  clade V | *Cel*SLO-1a | 1140 | 129.5 | NP_001024259 |
| *Caenorhabditis elegans* | Chromadorea  Rhabditida  Rhabditoidea  clade V | *Cel*SLO-1b | 1118 | 127.0 | NP_001024260 |
| *Caenorhabditis elegans* | Chromadorea  Rhabditida  Rhabditoidea  clade V | *Cel*SLO-1c | 1131 | 128.6 | NP_001024261 |
| *Caenorhabditis briggsae* | Chromadorea  Rhabditida  Rhabditoidea  clade V | *Cbr*SLO-1 | 1142 | 129.7 | XP_2638493 |
| *Caenorhabditis remanei* | Chromadorea  Rhabditida  Rhabditoidea  clade V | *Cre*SLO-1 | 1167 | 132.6 | XP_003094347 |
| *Haemonchus contortus* | Chromadorea  Rhabditida  Rhabditoidea  clade V | *Hco*SLO-1 | 1105 | 125.2 | ABS45068 |
| *Cooperia oncophora* | Chromadorea  Rhabditida  Rhabditoidea  clade V | *Con*SLO-1 | 1111 | 125.9 | ABS45069 |
| *Ancylostoma caninum* | Chromadorea  Rhabditida  Rhabditoidea  clade V | *Aca*SLO-1 | 1116 | 126.7 | ACC68842 |
| *Pristionchus pacificus* | Chromadorea  Rhabditida  Rhabditoidea  clade V | *Ppa*SLO-1 | 1188 | 134.1 | PPA29145^b^ |
| Species | Systematic position | Protein | Amino acid length | Molecular  Weight (kDa) | Accession no.^a^ |
| *Strongyloides ratti* | Chromodorea  [Rhabditida](http://www.ncbi.nlm.nih.gov/Taxonomy/Browser/wwwtax.cgi?mode=Undef&id=6236&lvl=3&lin=f&keep=1&srchmode=1&unlock)  [Panagrolaimoidea](http://www.ncbi.nlm.nih.gov/Taxonomy/Browser/wwwtax.cgi?mode=Undef&id=55746&lvl=3&lin=f&keep=1&srchmode=1&unlock)  Clade IV | *Sra*SLO-1 | 1170 | 131.4 | Sr321_0X0013600.t1^c^ |
| *Meloidogyne incognita* | Chromodorea  Tylenchida  Tylenchoidea  CladeIV | *MinSLO-1* | 1120 | 126.4 | Minc04076a^d^ |
| *Dirofilaria immitis* | Chromadorea  Spirurida  clade III | *Dim*SLO-1a | 1119 | 126.7 | AFH88396 |
| *Dirofilaria immitis* | Chromadorea  Spirurida  clade III | *Dim*SLO-1b | 1104 | 125.0 | AFX93730 |
| *Onchocerca gutturosa* | Chromadorea  Spirurida  clade III | *Ogu*SLO-1 | 1119 | 126.8 | ADY18306 |
| *Brugia malayi* | Chromadorea  Spirurida  clade III | *Bma*SLO-1c | 1115 | 126.3 | BM6719c^b^ |
| *Brugia malayi* | Chromadorea  Spirurida  clade III | *Bma*SLO-1d | 1132 | 128.2 | BM6719d^b^ |
| *Brugia malayi* | Chromadorea  Spirurida  clade III | *Bma*SLO-1e | 1079 | 122.3 | BM6719e^b^ |
| *Brugia malayi* | Chromadorea  Spirurida  clade III | *Bma*SLO-1f | 1104 | 125.1 | BM6719f^b^  KJ531222 |
| *Brugia malayi* | Chromadorea  Spirurida  clade III | *Bma*SLO-1g | 1087 | 123.2 | BM6719g^b^ |
| Species | Systematic position | Protein | Amino acid length | Molecular  Weight (kDa) | Accession no.^a^ |
| *Brugia malayi* | Chromadorea  Spirurida  clade III | *Bma*SLO-1h | 1104 | 125.0 | BM6719h^b^ |
| *Onchocerca ochengi* | Chromadorea  Spirurida  clade III | *Ooc*SLO-1a | 1119 | 126.7 | MW039265 |
| *Onchocerca ochengi* | Chromadorea  Spirurida  clade III | *Ooc*SLO-1b | 1119 | 126.7 | MW039266 |
| *Onchocerca volvolus* | Chromadorea  Spirurida  clade III | *Ovo*SLO-1a | 1119 | 126.8 | OVOC4127a^b^ |
| *Onchocerca volvolus* | Chromadorea  Spirurida  clade III | *Ovo*SLO-1b | 1119 | 126.8 | OVOC4127b^b^ |
| *Onchocerca volvolus* | Chromadorea  Spirurida  clade III | *Ovo*SLO-1c | 1104 | 125.1 | OVOC4127c^b^ |
| *Onchocerca volvolus* | Chromadorea  Spirurida  clade III | *Ovo*SLO-1d | 1143 | 125.1 | OVOC4127d^b^ |
| *Onchocerca volvolus* | Chromadorea  Spirurida  clade III | *Ovo*SLO-1f | 1119 | 129.5 | OVOC4127f^b^ |
| *Ascaris suum* | Chromadorea  Ascaridoidea  clade III | *Asu*SLO-1 | 1117 | 126.4 | ACC68842 |
| *Parascaris equorum* | Chromadorea  Ascaridoidea  clade III | *Peq*SLO-1 | 1108 | 125.2 | ACC68843 |
| Species | Systematic position | Protein | Amino acid length | Molecular  Weight (kDa) | Accession no.^a^ |
| *Toxocara canis* | Chromadorea  Ascaridoidea  clade III | *Tca*SLO-1 | 1123 | 126.9 | ACJ64718 |
| *Trichuris muris* | Enoplea  Trichocephalida  clade I | *Tmu*SLO-1.1a | 1151 | 130.1 | AEB96250 |
| *Trichuris muris* | Enoplea  Trichocephalida  clade I | *Tmu*SLO-1.1b | 1151 | 130.2 | KJ531218 |
| *Trichuris muris* | Enoplea  Trichocephalida  clade I | *Tmu*SLO-1.1c | 346 | 41.5 | KJ531219 |
| *Trichuris muris* | Enoplea  Trichocephalida  clade I | *Tmu*SLO-1.1d | 303 | 34.7 | KJ531220 |
| *Trichuris muris* | Enoplea  Trichocephalida  clade I | *Tmu*SLO-1.2 | 1123 | 126.9 | KJ531221 |
| *Trichinella spiralis* | Enoplea  Trichocephalida  clade I | *Tsp*SLO-1.1 | 1134 | 128.5 | XP_003370273  XP_003370274^e^ |
| *Trichinella spiralis* | Enoplea  Trichocephalida  clade I | *Tsp*SLO-1.2 | 1129 | 128.0 | XP_003370270  XP_003370271  XP_003370272^e^ |
| *Daphnia pulex* | Ecdysozoa  Crustacea  Branchiapoda | *Dpu*SLO-1 | 1028 | 115.7 | EFX85873 |
| *Pediculus humanus corporis* | Ecdysozoa  Insecta  Anoplura | *Phu*SLO-1 | 1141 | 127.9 | XP_002425826 |
| Species | Systematic position | Protein | Amino acid length | Molecular  Weight (kDa) | Accession no.^a^ |
| *Anopheles gambiae* | Ecdysozoa  Insecta  Diptera | *Aga*SLO-1 | 1154 | 129.1 | XP_313505.5 |
| *Drosophila melanogaster* | Ecdysozoa  Insecta  Diptera | *Dme*SLO-1Q | 1175 | 130.3 | NP_001014651 |
| *Drosophila melanogaster* | Ecdysozoa  Arthropoda  Diptera | *Dme*SLO-1S | 1210 | 134.4 | NP_001163712 |
| *Aplysia californica* | Lophotrochozoa  Mollusca  Gastropoda | *Acal*SLO-1 | 1070 | 120.2 | AAR27959 |
| *Gallus gallus* | Vertebrata  Aves | *Gga*SLO-1 | 1140 | 128.1 | AAD16633 |
| *Bos taurus* | Vertebrata  Mammalia  Ruminantia | *Bta*SLO-1 | 1166 | 130.1 | NP_777105 |
| *Homo sapiens* | Vertebrata  Mammalia  Primates | *Hsa*SLO-1 | 1236 | 135.6 | NP_001154824 |

^a^If not further specified protein accession numbers from GenBank are given.

^b^In WormBase.

^c^In Sanger *S. ratti* genome database.

^d^In INRA *M. incognita* genome database.

^e^A full-length consensus sequence was built from these entries.
