# Supplementary material for: Development of emodepside as a possible adulticidal treatment for human onchocerciasis—The fruit of a successful industrial–academic collaboration
Source: PLoS Pathog. 2021 Jul 22;17(7):e1009682. doi: 10.1371/journal.ppat.1009682 (PMC8297762; doi:10.1371/journal.ppat.1009682)
Supplement: S2 Table — (DOCX) [file ppat.1009682.s004.docx]

**Efficacy studies for emodepside against *Trichuris* spp., ascarids, hookworms and *Strongyloides ratti*.**

| **Emodepside treatment** | **Species investigated** | **Activity** | **Reference** |
| --- | --- | --- | --- |
| 3 consecutive oral doses (0.1–100 mg/kg) | *Trichuris muris* (mouse) | ED_95_ (WBR) 24.5 mg/kg | [103] |
| 3 consecutive intraperitoneal doses (10–250 mg/kg) | *Trichuris muris* (mouse) | ED_95_ (WBR) 40.0 mg/kg | [103] |
| 3 consecutive subcutaneous doses (50–500 mg/kg) | *Trichuris muris* (mouse) | ED_95_ (WBR) 40.7 mg/kg | [103] |
| Single oral dose (1.25–10 mg/kg and 75 mg/kg) | *Trichuris muris* (mouse) | 100% WBR at 75 mg/kg; ED_50_ (WBR) 1.2 mg/kg | [104] |
| 0.45 mg/kg oral | *Trichuris vulpis* (dog) | 100% WBR | [37] |
| 1 mg/kg (plus praziquantel) oral | *Trichuris vulpis* (dog) | 99.9% ERR | [34] |
| 0.5, 1, 2 mg/kg oral | *Trichuris vulpis* (dog) | >99% WBR | [39] |
| Spot-on (2.14% (w/w) emodepside and 8.58% (w/v) praziquantel) ≥ 3 mg/kg emodepside | *Toxocara cati* (cat) | 96.8%, ≥99.4% and 100% WBR against L3, L4, and adults | [38] |
| Spot-on (2.14% (w/w) emodepside and 8.58% (w/v) praziquantel) ≥3 mg/kg emodepside | *Toxascaris leonina* (cat) | ≥93.4% WBR | [38] |
| 1 mg/kg (plus praziquantel) oral | *Toxocara canis* (dog) | >94%, >98%, >92% and >99% WBR against L3, L4, and immature and mature adults | [34] |
| 1 mg/kg (plus praziquantel) oral | *Toxascaris leonina* (dog) | >95%, >99% and >99% WBR against L4 and immature and mature adults | [34] |
| 0.45 mg/kg (plus toltrazuril) oral | *Toxocara canis* (dog) | 99.3%, ≥94.7, 100% WBR against L4, and immature and mature adults | [108] |
| Spot-on (2.14% (w/w) emodepside and 8.58% (w/v) praziquantel) ≥3 mg/kg emodepside | *Ancylostoma tubaeforme* (cats) | >95% and >97% WBR against L4 and immature adults, 100% WBR against mature stages | [33] |
| **Emodepside treatment** | **Species investigated** | **Activity** | **Reference** |
| 1 mg/kg (plus praziquantel) oral | *Ancylostoma caninum* (dog) | >98% WBR against immature and mature stages | [39] |
| 0.45 mg/kg (plus toltrazuril) oral | *Ancylostoma caninum* (dog) | ≥99.5 % WBR against adults | [108] |
| 0.1 mg/kg, 0.25 mg/kg (plus toltrazuril (2%) oral | *Ancylostoma tubaeforme* (cats) | Worm expulsion rates of 95.7 and 100% | [37] |
| Recommended dose (>3 mg/kg) (plus praziquantel) spot on | *Ancylostoma ceylanicum* (cat) | 100% ERR | [109] |
| 2.5 mg/kg oral | *Ancylostoma ceylanicum* (hamster) | 100% WBR | [104] |
| Single oral dose (1.25–10 mg/kg) | *Necator americanus* (hamster) | 100% WBR at 5 and 10 mg/kg; ED_50_ 0.5 mg/kg | [104] |
| 1–10 mg/kg oral | *Heligmosomoides bakeri* (mouse) | 5 and 10 mg/kg 100% WBR against adult stages | [107] |
| 1–10 mg/kg | *Nippostrongylus brasiliensis* (rat) | 2.5, 5 and 10 mg/kg resulted in 0% survival rate when treated between 4 and 8 days post infection | [107] |
| 1–10 mg/kg oral | *Strongyloides ratti* (rat) | 5 and 10 mg/kg resulted in 0% survival rate when treated between 2 and 9 days post infection | [107] |

Worm burden reduction (WBR); egg reduction rate (ERR); w/w, weight per weight; w/v, weight per volume; L3, third-stage larvae; L4, fourth-stage larvae.
